# Modeling Human Protein Physical Interactions Involved in HIV Attachment In Silico

**DOI:** 10.3390/ijms262211209

**Published:** 2025-11-20

**Authors:** Vladimir S. Davydenko, Alexander N. Shchemelev, Yulia V. Ostankova, Ekaterina V. Anufrieva, Areg A. Totolian

**Affiliations:** Saint Petersburg Pasteur Institute, 197101 St. Petersburg, Russia; vladimir_david@mail.ru (V.S.D.); shenna1@yandex.ru (Y.V.O.); kate.an21@yandex.ru (E.V.A.); totolian@spbraaci.ru (A.A.T.)

**Keywords:** human immunodeficiency virus, virus–host interaction, protein-protein interactions, candidate genes, in silico, CD4, CCR5, CXCR4, CCR2, computer modeling, AlphaFold, ChimeraX

## Abstract

The human immunodeficiency virus (HIV) remains a major global health challenge. A promising therapeutic strategy involves identifying human proteins capable of physically blocking viral entry by interacting with key components of the HIV attachment system. To address this challenge systematically, we developed a computational pipeline for prioritizing protein–protein interaction and applied it to identify host proteins interacting with the viral glycoprotein gp120 and cellular receptors (CD4, CCR5, CXCR4, CCR2). Our approach combined large-scale interaction modeling using AlphaFold 3 with a comprehensive comparative analysis framework. We screened a panel of 55 candidate human proteins selected through integrated bioinformatics analysis. The pipeline incorporated model confidence assessment, quantitative contact analysis, and normalization against reference interactions to generate a robust ranking of candidates. Key findings reveal several important patterns. Chemokine CCL27 uniquely demonstrated high binding potential to both CCR5 co-receptor and viral gp120, suggesting its potential for dual-blockade capability. Analysis of natural ligand interactions with chemokine receptors showed marked disparity: CC-chemokine family members exhibited significantly greater binding capacity for CCR5 and CCR2 receptors compared to CXC-family ligand interactions with CXCR4. This binding imbalance may potentially drive selective viral pressure and influence tropism evolution during disease progression. We also identified potential interactions between HIV entry components and neuropeptides including PNOC and NPY, as well as various membrane receptors beyond classical coreceptors. Furthermore, cluster analysis revealed clear separation between receptor-type and ligand-type interactors, supporting the biological plausibility of our predictions. While acknowledging limitations related to model refinement, this study provides a systematically ranked set of candidate targets for HIV therapeutic development. Beyond identifying specific HIV interaction candidates, this study establishes a generalizable computational pipeline for the prioritization of protein–protein interaction in pathogen-host systems, effectively bridging large-scale modeling.

## 1. Introduction

The human immunodeficiency virus (HIV) remains one of the most significant global health challenges [1]. Despite advances in antiretroviral therapy (ART) targeting key stages of the viral replication cycle [2], infection persistence is largely driven by the emergence of drug-resistant viral mutations, reducing the efficacy of existing treatment regimens [3,4]. This underscores the urgent need to discover new bioactive compounds and therapeutic strategies to overcome current protocol limitations.

HIV research relevance extends beyond developing drugs targeting various viral cycle stages to identifying novel molecular targets and endogenous infection control mechanisms. This necessity stems from the extreme complexity of virus–human immune system interactions [5], which significantly constrains existing therapeutic strategies’ effectiveness [6,7]. A promising direction involves identifying endogenous innate immunity factors with antiviral activity. Studies demonstrate that certain human proteins, like chemokine CCL3, can inhibit infection by binding chemokine co-receptors and competitively blocking viral entry [8]. Crucially, such inhibition targets cellular rather than viral components. Key advantages of these endogenous inhibitors include their preexistence in the human body, partially understood physiological roles and safety profiles, and known interaction mechanisms with potential target proteins for some. A prominent example is APOBEC3G, which inhibits HIV-1 replication by inducing lethal hypermutation in newly synthesized viral DNA, thereby blocking reverse transcription and integration processes [9]. However, the systematic identification of such endogenous inhibitors among broad panels of host proteins remains challenging.

Current approaches for interaction discovery include public databases enable reconstruction of large-scale protein–protein interaction (PPI) networks. Such network analysis helps identify key protein hubs associated with HIV pathogenesis for further in-depth study [10]. However, these networks often reflect functional associations or co-expression rather than direct physical interaction, potentially yielding false-positive results. Even for physical interactions, network analysis typically lacks molecular mechanism details, spatial characteristics (complex conformation and stoichiometry), binding affinity, or process dynamics. Identifying physical interactions involving direct atom-atom contact between molecules is critical, as they underpin most fundamental cellular processes: signal transduction, enzymatic catalysis, viral particle assembly, and function blocking. Therefore, establishing physical contact between host proteins and viral proteins and/or established cellular cofactors opens avenues for designing targeted low-molecular-weight inhibitors or peptidomimetics that can specifically disrupt this interaction, thereby interrupting the pathogen’s life cycle. Simultaneously, this research direction deepens understanding of molecular pathogenesis mechanisms by revealing key target proteins and regulatory pathways involved in disease development. However, conventional PPI networks typically cannot distinguish between direct physical interactions and indirect functional associations.

The comprehensive experimental determination of physical interactions across the full spectrum of potential host–pathogen protein pairs presents substantial practical challenges due to the exceptional resource intensity involved. Such large-scale experimental screening demands immense investments of time, specialized equipment, and materials, rendering exhaustive experimental approaches impractical for initial candidate discovery. In this context, preliminary computer modeling offers a powerful strategy to optimize the search process through sophisticated in silico assessment. Modern computational methods can generate rationally justified priority target lists for subsequent experimental validation, thereby concentrating research efforts on the most promising candidates. Recent advances in structural bioinformatics, particularly the development of AlphaFold and related deep learning architectures [11], have revolutionized our ability to predict protein–protein interaction with unprecedented accuracy. These approaches enable detailed characterization of binding interface formation between viral and cellular proteins at atomic resolution, predicting spatial architecture of complexes and identifying specific amino acid residues that dominate binding energy contributions. The resulting structural insights provide critical foundation for multiple downstream applications, including virtual screening of low-molecular-weight compound libraries to identify candidates capable of sterically or allosterically disrupting pathogenic complex formation. However, the efficient processing and prioritization of hundreds of predicted complexes generated by these methods require specialized analytical pipelines. Thus, despite the power of computational modeling, a pressing need remains for integrated frameworks that can systematically evaluate and rank large volumes of predicted protein complexes to maximize research efficiency [12,13,14]. The primary aim of this study was to develop a computational pipeline for predicting protein–protein interaction and to apply it to identify human proteins capable of physically interacting with the viral glycoprotein gp120 and/or major HIV cellular co-receptors (CD4, CCR5, CXCR4, CCR2).

## 2. Results

The study proceeded through several key stages: initial validation of protein structures, modeling of binary interactions, quantitative analysis of interfaces, and finally, a comparative ranking of candidates based on a composite metric. The results of each stage are detailed below.

To validate the modeling workflow, we first generated single-protein structures for each host receptor and the HIV gp120 glycoprotein (five single models of HCBGPs/HRP and one CCR5-Δ32 model). The parameters of these models are presented in Table 1. The obtained protein structures visually correspond to models available in protein databases, while pTM and RS parameter values indicate close approximation to the native structures of the analyzed proteins.

The subsequent stage involved modeling interactions between receptors/coreceptors and the HRP (5 models). Model parameters are presented in Table 2.

**Figure 1:**
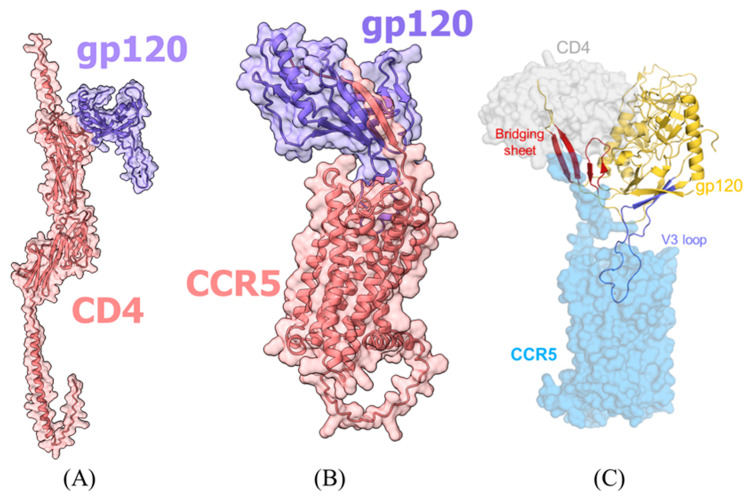
Correspondence between predicted interaction models for CCR5+gp120 and CD4+gp120 with experimental data. Panels: (**A**) AlphaFold CD4 (pink)+gp120 (purple); (**B**) AlphaFold CCR5 (pink)+gp120 (purple); (**C**) representation of CCR5, CD4, and gp120 interaction based on experimental data [15].

In line with the expected limitations of our simplified modeling approach (Section 4.2.4), the generated models for biologically established reference complexes did not achieve high interface confidence values (ipTM < 0.6 for all HCBGP-HRP pairs), despite their plausible visual appearance and correspondence with known interaction patterns (Figure 1). This consistent result across all reference pairs confirms that absolute ipTM scores are not reliable discriminators in this specific screening context. It thereby reinforces the validity of our decision to employ a comparative analysis framework based on the composite area metric, which normalizes predicted interactions against these internal reference benchmarks.

We generated interaction models for each background protein paired with every candidate protein, resulting in a total of 275 protein–protein interaction models. Model confidence results are presented in Appendix A. Among these, 68 models showed reliable pTM but unreliable RS, while 37 models demonstrated both reliable RS and pTM but unreliable ipTM. Two models (ADRA2C, FPR3) exhibited reliable RS but unreliable ipTM and pTM.

**Figure 2:**
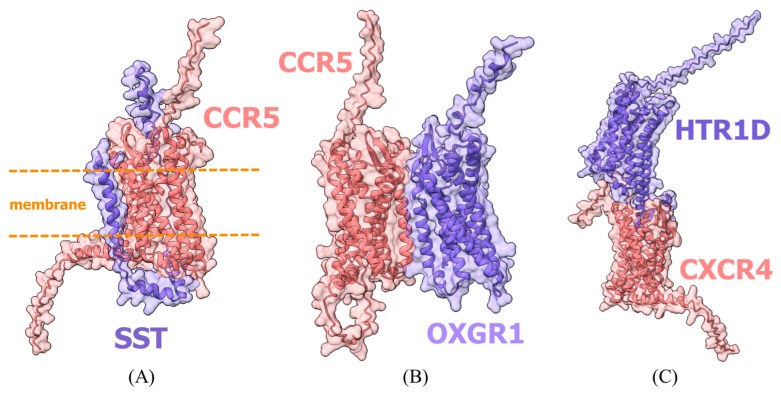
Examples of primary errors in protein interaction modeling. Panels: (**A**) protein contacts both receptor termini, effectively penetrating the cell membrane, SST (purple) + CCR5 (pink); (**B**) receptors positioned adjacent to each other, OXGR1 (purple) + CCR5 (pink); (**C**) receptors stacked vertically, HTR1D (purple) + CXCR4 (pink). This conformation is possible but highly improbable.

It should be noted that despite satisfactory confidence metrics for several predicted models, physical interactions between these proteins in the presented conformations remain unlikely due to steric constraints and electrostatic incompatibility (Figure 2). While these artifacts represent known limitations of the simplified docking system, we intentionally retained all models in subsequent analysis to demonstrate the pipeline’s ability to handle diverse prediction scenarios. A visual assessment of model plausibility was performed, though this evaluation necessarily remains subjective; its results are provided in Appendix B.

Having established a set of complex models, we proceeded to quantitatively characterize the protein-protein interfaces. We analyzed atomic contacts, steric clashes, and hydrogen bonds to derive a normalized interaction score (I_n_) for each candidate complex. This provided a biophysical characterization of the interaction interfaces, complementing the structural confidence metrics. The resulting contact data for the background genes are presented in Table 3.

Contact data for HICGP interactions with each background protein and their normalized metrics are presented in Appendix C, Table A3 and Table A4.

**Figure 3:**
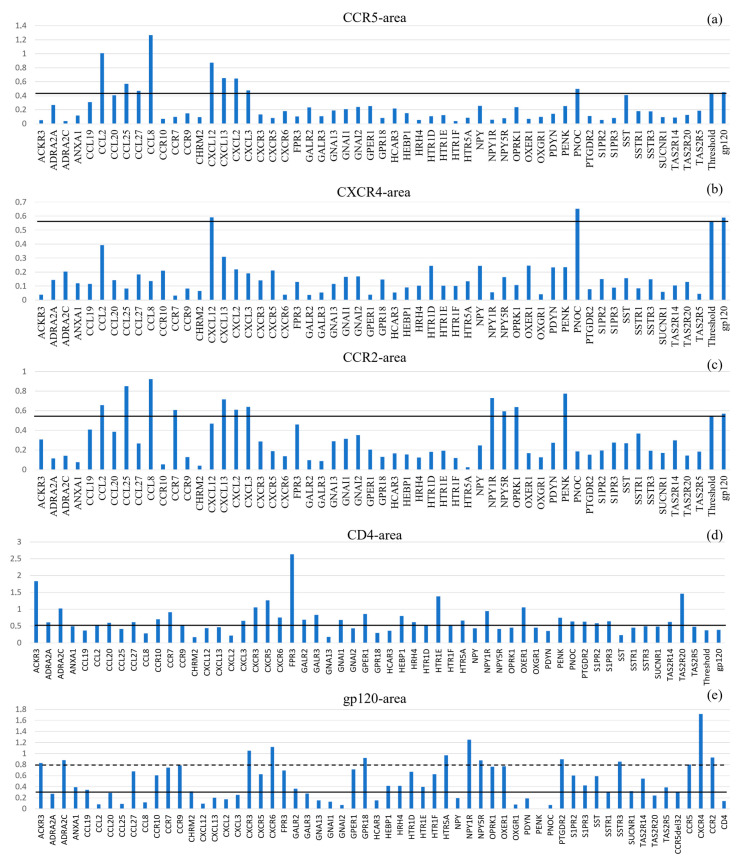
Comparative analysis of candidate interactions based on the composite metric Normalized Interaction Area (A = RS × I_n_), which integrates model confidence (Ranking Score, RS) and normalized interface contact count (I_n_). Panels: (**a**) Interaction areas with CCR5; (**b**) with CXCR4; (**c**) with CCR2; (**d**) with CD4; (**e**) Interaction areas between gp120 and candidate/background proteins. For upper panels (**a**–**d**), the solid line represents the prioritization threshold based on the gp120+HCBGPs area. For panel (**e**), the solid line represents the threshold based on the gp120+CCR5Δ32 area; the dashed line represents the gp120+CCR5 area.

To integrate both the model confidence (RS) and the interface quality (I_n_) into a single prioritization metric, we calculated the composite Normalized Interaction Area (A = RS × I_n_). This approach allowed for the systematic ranking of all candidate interactions against our internal reference set (gp120-HCBGPs). The resulting landscape of interaction areas revealed distinct clusters and high-priority candidates (Figure 3, Table 4).

For standardization, interactions between HICGPs and background proteins were considered significant when their area values exceeded 95% of the area value for the corresponding background protein’s interaction with gp120 (excluding the gp120 interaction itself). Complete calculation data are presented in Appendix D and Appendix E. Table 4 presents HICGPs with the most significant area values.

Significant proteins based on calculated interaction area with CCR5 were: CCL2, CCL25, CCL27, CCL8, CXCL12, CXCL13, CXCL2, CXCL3, and PNOC. Significant proteins based on calculated interaction area with CXCR4 were: CXCL12 and PNOC. Significant proteins based on calculated interaction area with CCR2 were: CCL2, CCL25, CCL8, CCR7, CXCL13, CXCL2, CXCL3, NPY1R, NPY5R, OPRK1, and PENK.

Analysis of CD4-gp120 interactions reveals a limited number of atomic contacts and insignificant binding surface area. Consequently, the diagnostic value of this complex for comparative analysis is substantially reduced, as most investigated HICGPs demonstrate area values comparable to or exceeding that of the CD4+gp120 system. Similarly to the CD4+gp120 complex, the area in the gp120+CCR5-Δ32 system also proved insufficient for use as a threshold to exclude HICGPs with low significance. When considering the interaction area threshold of CCR5 with gp120, the candidate list is as follows: CCL27, CCR7, NPY1R, NPY5R, OPRK1, ACKR3, ADRA2C, CCR10, CCR9, CXCR3, CXCR5, CXCR6, FPR3, GPER1, HTR1D, HTR1F, HTR5A, OXER1, PTGDR2, S1PR2, SSTR3, TAS2R14, GPR18, and SST.

The primary proteins interacting with gp120 are receptors, a finding which is further supported by cluster analysis results presented in Table 5. A clear separation into receptor and ligand clusters is evident. Particular attention should be given to HICGP interaction models with HCBGPs/HRP that demonstrated area values exceeding established thresholds. No candidate proteins interacting with all background human proteins analyzed in this study were identified. HICGPs interacting with three HCBGPs (CCR5, CXCR4, CD4) were identified: PNOC and CXCL12. HICGPs interacting with one of the main coreceptors (CCR5 or CXCR4) and one or two other HCBGPs (CCR2 and/or CD4) were identified: CCL2, CCL8, CCL25, CCL27, CXCL13, CXCL3, and CXCL2. Interaction models are presented in files in the Supplementary Materials.

**Figure 4:**
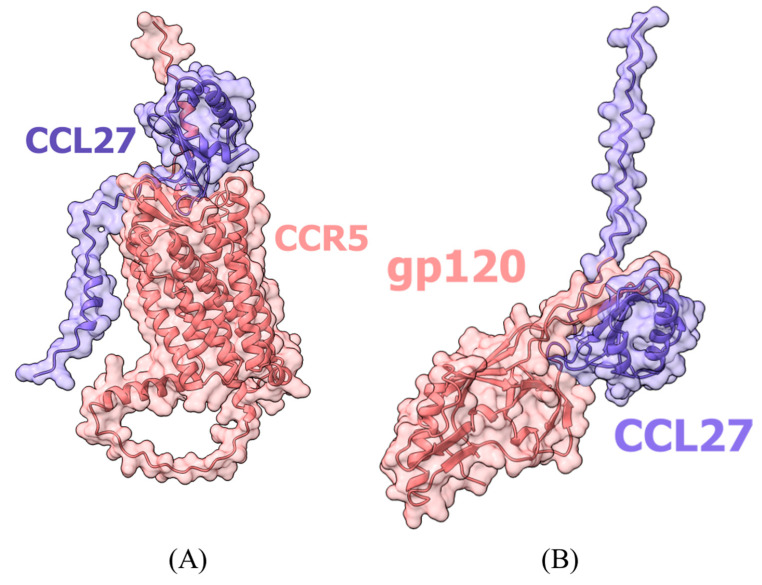
Interaction models with CCL27: (**A**) CCL27 with CCR5; (**B**) CCL27 with gp120. CCL27 represents the most promising candidate showing dual-binding potential, with high confidence scores across multiple metrics (RS = 0.69 with CCR5, 0.47 with gp120).

Among HICGPs, only CCL27 showed area values above thresholds for interaction models with both CCR5 and gp120 (Figure 4). Interaction models are presented in files in the Supplementary Materials.

## 3. Discussion

### 3.1. Overview of the Computational Approach and Key Findings

Top-ranked HICGPs based on comparative Area analysis (values exceeding operational thresholds for prioritization are indicated in bold). This study aimed to conduct an in silico screen of a panel of 55 HICGPs to identify molecules potentially capable of modulating a key stage of the HIV life cycle, namely the interaction between viral glycoprotein gp120 and cellular receptors [16]. The application of the AlphaFold 3 algorithm enabled the reconstruction and comprehensive analysis of 275 molecular complexes, resulting in the identification of both expected and previously undescribed potential targets for therapeutic intervention in HIV infection.

### 3.2. Validation of the Pipeline: Separation of Ligands and Receptors

The clear separation of candidate proteins into distinct receptor-type and ligand-type clusters, as revealed by our k-means cluster analysis (Table 5), provides internal validation for our computational pipeline and prioritization strategy. This recapitulation of fundamental biological categories demonstrates that our comparative framework, based on the composite Area metric, effectively captures biologically relevant features of protein–protein interaction. The clear dichotomy suggests that the predicted interaction models respect basic biological principles, where ligands (such as chemokines and neuropeptides) and receptors occupy distinct functional and structural niches, even within the simplified in silico environment. This successful separation reinforces the biological plausibility of the top-ranking candidates identified by our screening approach and supports the robustness of our method in distinguishing between different modes of potential interaction with the HIV entry machinery.

### 3.3. Chemokine Ligands: Expected and Discordant Results

As anticipated based on their known biological functions, C-C family chemokines (CCL2, CCL8, CCL25, CCL27) and C-X-C family chemokines (CXCL12, CXCL13, CXCL2, CXCL3) demonstrated high interaction potential with their natural receptors CCR5 and CCR2 in our models. Although normalized contact parameters for most did not exceed those of the gp120-coreceptor complexes, their binding capacity suggests these chemokines may act as natural competitive antagonists, potentially blocking viral glycoprotein binding sites.

Of particular note is the case of chemokine CCL2. Our model predicts high-affinity binding to CCR5, suggesting a potential for direct competitive inhibition of viral entry. This finding, however, appears to contradict experimental studies reporting that CCL2 can enhance HIV replication in vivo and ex vivo [17,18]. We propose that this discrepancy underscores the distinction between a direct physical interaction, captured by our structural models, and a protein’s net biological effect within a complex physiological environment.

The primary physiological role of CCL2 is the chemotaxis of monocytes and other immune cells to sites of inflammation [19]. This recruitment significantly expands the pool of target cells (e.g., CD4+ T cells, macrophages) available for HIV infection, an indirect proviral effect that likely dominates the net outcome in many experimental and physiological contexts. Thus, the in silico prediction and experimental observations can be reconciled within a dual-activity framework: CCL2 may possess an intrinsic, direct antiviral potential via coreceptor blockade (as predicted by our model), which is masked in vivo by its potent, indirect proviral effect via target cell recruitment.

This case highlights a critical principle for interpreting computational screens: a predicted physical interaction signifies mechanistic potential, but the net biological outcome is determined by the broader cellular and systemic context [20].

Analysis revealed a significant disproportion: the relative number of ligands interacting with CXCR4 was substantially lower than with CCR5/CCR2. These findings suggest that within the employed model, CC-type chemokines exhibit more pronounced inhibitory activity against viral utilization of CCR5 co-receptor. The observed binding disparity is consistent with the hypothesis that an imbalance in available natural ligands could contribute to selective pressures influencing coreceptor switching and the emergence of CXCR4-tropic variants in later stages of infection [18,21].

### 3.4. Potential gp120 Interactions with Non-Canonical Receptors

Beyond the classical co-receptors, our modeling suggests the capacity of the viral glycoprotein gp120 for direct interaction with a broad spectrum of cellular membrane receptors. Although overall prediction reliability was moderate—as expected given gp120’s high conformational plasticity and the challenges of predicting its binding sites—our comparative analysis identified two significant subgroups of potential interactors.

The first group comprises chemokine superfamily receptors (CCR10, CCR7, CCR9, CXCR3, CXCR5, CXCR6), which demonstrated contact quantities with gp120 comparable to reference coreceptors. These findings align with publications suggesting that some of these receptors may serve as alternative or supplementary viral entry portals into specific cell types [22,23]. The specific targeting of this receptor class by gp120 may represent a viral adaptation to broaden cellular tropism.

The second, more diverse group consisted of various neuroreceptors and other membrane proteins (HRH4, HTR1D, HTR1F, HTR5A, NPY1R, NPY5R, OPRK1, OXER1, OXGR1, PTGDR2, S1PR2, S1PR3, SSTR1, SSTR3, SUCNR1). For several of these, existing data suggest possible associations with HIV-associated neuropathologies [24], making them promising targets for further investigation in the context of neuroinvasion and neuropathogenesis.

Somatostatin (SST) merits particular attention in this context, as its expression level has been reported to correlate with HIV progression [25,26], although earlier studies refuted its substantial role [27], indicating ambiguity in existing data that warrants further clarification.

The G-protein family (GNAI1, GNAI2, GNA13) exhibited high structural confidence values (pTM) but low contact quantities with coreceptors, which is expected since they typically interact with receptor cytoplasmic domains. Their potential influence on infection is likely mediated through complex intracellular signaling cascades and cannot be adequately assessed within our binary interaction modeling methodology [28].

### 3.5. Hypothesis-Generating Predictions for Neuropeptides

The high ranking of several neuropeptides (PNOC, NPY, PDYN, PENK) among the candidate interactors warrants specific discussion. Their prioritization should be interpreted with particular caution due to inherent methodological considerations. The small size and inherent structural flexibility of neuropeptides pose a particular challenge for reliable modeling using static docking approaches, potentially allowing for multiple conformations and leading to overestimated confidence in some binding poses. Indeed, some models showed potential binding to sterically inaccessible sites, such as intracellular domains. Therefore, while these candidates ranked highly in our screen, they should be classified as the most speculative predictions, serving primarily to generate hypotheses for rigorous experimental validation.

Notably, the literature analysis provides indirect support for the potential biological relevance of these systems in the context of HIV infection. For instance, anterior cingulate cortex samples from Patients with HIV showed decreased PDYN (prodynorphin) gene mRNA levels alongside increased OPRK1 (kappa-opioid receptor) mRNA expression compared to controls [24]. We hypothesize that reduced PDYN expression may represent a compensatory mechanism aimed at limiting monocyte recruitment and mitigating neuroinflammatory processes, while enhanced OPRK1 expression might be associated with attempts to modulate proinflammatory signaling pathways. Furthermore, increased neuropeptide Y (NPY)-like immunoreactivity has been observed in the cerebrospinal fluid of Patients with HIV, suggesting a potential link to HIV encephalopathy [29]. Thus, while the direct physical interactions predicted by our models remain highly speculative, the involved neuropeptide systems appear to be engaged in the host response to HIV infection, particularly within the nervous system.

### 3.6. Limitations of the Study

While our computational pipeline provides a systematic approach for prioritizing protein interactions, several limitations should be acknowledged. First, the low ipTM scores observed for biologically validated complexes are a direct consequence of our simplified binary modeling strategy, which traded atomic-level refinement for screening throughput. This inherent trade-off is why our comparative analysis framework, rather than absolute confidence scores, forms the core of the prioritization pipeline. Second, our models represent simplified binary interactions without key biophysical contexts such as explicit lipid membranes, gp120 glycosylation, or physiological ionic conditions. Third, the operational thresholds and confidence intervals used for candidate selection were derived from a comparative analysis with biologically verified reference interactions rather than from rigorous statistical distributions of null models. While this practical approach allowed for large-scale prioritization, it lacks a formal statistical foundation. Finally, and most importantly, all predictions—particularly those involving neuropeptides and novel receptor interactions—require experimental validation (e.g., SPR, BLI, cellular assays) before any firm biological conclusions can be drawn. We reiterate that the term “significant” throughout this manuscript refers specifically to candidates that surpassed our operational, comparative thresholds for prioritization within this computational screen. These thresholds provide a systematic ranking for guiding future research but do not constitute statistical or biological validation of the interactions.

### 3.7. Concluding Remarks

This in silico study successfully demonstrates a generalizable computational pipeline for the prioritization of protein–protein interaction in pathogen-host systems. By applying this framework to HIV-1 attachment, we have systematically narrowed a broad panel of candidates to a focused set of high-priority targets. Our results not only recapitulate known biology, validating our approach, but also generate novel and sometimes unexpected hypotheses regarding viral engagement with chemokine and neuromodulatory systems. The predictions presented here, most notably the dual-binding candidate CCL27, establish a robust and prioritized foundation for guiding future experimental efforts aimed at validating these interactions and exploring their therapeutic potential. This work underscores the power of integrated computational modeling to illuminate complex host–pathogen interaction landscapes.

## 4. Materials and Methods

### 4.1. Materials for Modeling Physical Interactions

The analysis utilized a panel of 55 candidate proteins selected using our previously developed integrated scoring system [30]. This system weighted gene-disease associations based on tissue-specific expression levels, subcellular localization, biological pathway annotations, and participation in relevant biological processes. The study included key cellular receptors associated with HIV entry (CCR5, CXCR4, CCR2, CD4) [31,32] and the viral glycoprotein gp120, which mediates initial virus-target cell binding [16]. The scoring algorithm assigned greater weight to associations with the primary co-receptors CCR5 and CXCR4.

Primary amino acid sequences were obtained from the NCBI database for: HIV-interacting candidate gene proteins, HICGPs (ACKR3, ADRA2A, ADRA2C, ANXA1, CCL19, CCL2, CCL20, CCL25, CCL27, CCL8, CCR10, CCR7, CCR9, CHRM2, CXCL12, CXCL13, CXCL2, CXCL3, CXCR3, CXCR5, CXCR6, FPR3, GALR2, GALR3, GNA13, GNAI1, GNAI2, GPER1, GPR18, HCAR3, HEBP1, HRH4, HTR1D, HTR1E, HTR1F, HTR5A, NPY, NPY1R, NPY5R, OPRK1, OXER1, OXGR1, PDYN, PENK, PNOC, PTGDR2, S1PR2, S1PR3, SST, SSTR1, SSTR3, SUCNR1, TAS2R14, TAS2R20, TAS2R5); HIV coreceptor background gene proteins, HCBGPs (CCR5, CXCR4, CCR2, CD4); and the HIV reference protein, HRP (gp120). The complete sequence list appears in Appendix F.

We selected a CCR5-tropic gp120 variant for modeling, with its amino acid sequence corresponding to the HIV subtype A6 isolate predominant in Russia, ensuring relevance to regional epidemiological characteristics. The analysis incorporated the Δ32 nonsense mutation (CCR5-del32), which causes a 32-base pair deletion in the CCR5 gene resulting in a truncated, non-functional protein. Physical interaction modeling included this structurally modified CCR5 protein form. Computational analysis employed an in silico reconstructed CCR5 amino acid sequence featuring the specific deletion, enabling assessment of structural alterations on protein–protein interaction interfaces.

### 4.2. Physical Protein–Protein Interaction Modeling

To address the study objectives, we reconstructed three-dimensional structures for all specified proteins. Subsequent interaction modeling between HICGPs and HCBGPs/HRP employed the AlphaFold 3 algorithm (https://alphafoldserver.com/, developed by Google DeepMind, London, UK, accessed on 17 October 2025), recognized as one of the most effective tools for in silico protein structure reconstruction. This algorithm demonstrates high accuracy in predicting individual polypeptide structures and enables heterodimeric protein complex prediction, albeit with slightly reduced confidence compared to monomeric predictions [11].

The modeling results enabled candidate protein filtering, selecting only those forming statistically significant complexes with target structures for further investigation. Constructed models included confidence metrics for each protein complex. We utilized the highest-confidence model for subsequent analysis. To standardize the research approach, molecular modeling assessed interactions between HCBGPs and the viral glycoprotein gp120 (HRP). The same in silico approach analyzed the CCR5Δ32 variant receptor containing the Δ32 deletion, which served to establish computational validation thresholds.

#### 4.2.1. Model Confidence Assessment

The initial analysis stage involved evaluating model reliability. We assessed output data quality using predicted template modeling (pTM) and interface predicted template modeling (ipTM) metrics. These parameters were evaluated both individually and as components of the composite ranking score (RS), calculated according to Equation (1).

(1) $$RS=0.8\times ipTM+0.2\times pTM+0.5\times d-100\times N_{c};$$

wherein RS—ranking score;

pTM—predicted template modeling;

ipTM—interface predicted template modeling;

d—disorders;

*N*_c_—number of clusters.

According to the AlphaFold manual, a pTM score below 0.5 generally indicates that the predicted model may not closely resemble the true structure. Similarly, ipTM values below 0.6 suggest potentially unreliable modeling of the protein-protein interface region. The RS serves as a unified metric, where values ≥ 0.5 indicate models with acceptable reliability [11]. These RS values were utilized for all subsequent analyses.

As anticipated for highly flexible and context-dependent systems like the gp120 glycoprotein, which requires CD4 binding for its mature co-receptor interaction state [15,33], even reference complexes with established biological validity (e.g., gp120-CD4, gp120-CCR5) did not achieve high ipTM scores (>0.6) in our simplified binary modeling approach. This expected outcome directly reflects our methodological choice to prioritize large-scale screening over modeling complex biophysical contexts and underscores the necessity of our comparative framework, which relies on relative rather than absolute confidence metrics for candidate prioritization.

#### 4.2.2. Analysis of Contact Quantification in Predicted Models

The next stage involved analyzing contacts in the obtained structures. Using ChimeraX software v1.10.1, developed by the Resource for Biocomputing, Visualization, and Informatics (San Francisco, CA, USA) [34], we analyzed three categories: contacts, clashes, and hydrogen bonds. The total number of protein interactions (Interaction) was calculated as follows (Equation (2)):

(2) $$I=N_{ct}-N_{c}+N_{h};$$

wherein I—interactions;

N_ct_—numbers of contacts;

N_c_—numbers of clashes;

N_h_—numbers of H-bonds.

This metric penalizes non-physical steric clashes while rewarding specific favorable interactions (hydrogen bonds), providing a more biophysically meaningful estimate of interface quality compared to our initial formulation.

For HICGP interactions with background proteins (HCBGPs), the obtained data were normalized against the interaction count (I_n_) between that specific background protein and gp120. For HICGP interactions with gp120 (HRP), normalization was performed using the gp120-CCR5Δ32 interaction count, representing the minimal observed interaction value. It should be noted that gp120 affinity for CCR5 increases due to conformational changes induced by viral protein interaction with CD4 [15,33]. However, reliable computational modeling of such three-protein complexes remains challenging [11]. The per-residue confidence scores (pLDDT) for all predicted models are provided in the Supplementary Materials. While these data offer valuable granular insight into local model quality, they were not incorporated into our primary ranking pipeline due to the scale of the study. Our comparative framework was designed to utilize global and interface-level metrics (pTM, ipTM, RS) for high-throughput prioritization. The provided pLDDT data are intended for researchers who wish to perform a more detailed, residue-level assessment of the specific models of interest identified by our screen.

#### 4.2.3. Comprehensive Model Comparison Based on Derived Parameters

For comprehensive model comparison, the resulting set of models was plotted on a coordinate plane with axes corresponding to RS and I_n_. The integrative metric, accounting for both parameters, was selected as the area (A) of the rectangle formed between the coordinate axes and the perpendicular distances from the data point to these axes (Equation (3)).

(3) $$A=RS\times I_{n};$$

wherein A—area;

RS—ranking score;

I_n_—normalized Interaction.

To establish a robust operational threshold for candidate prioritization, we defined a cutoff at 95% of the Area value for each background protein’s interaction with gp120. This threshold was selected based on established practices in biological screening and aligns with the standard statistical significance level (*p* < 0.05) widely adopted in biomedical sciences [35]. This approach creates a normalized, comparative benchmark derived from the internal reference set of known biological interactions (gp120-HCBGPs), rather than relying on arbitrary absolute thresholds. By using the 95th percentile, we aim to select candidates whose composite interaction score (A = RS × I_n_) is nearly equivalent to or exceeds that of our positive controls, ensuring a high degree of confidence for subsequent experimental validation. For interactions with gp120 itself, we analyzed all Area values exceeding that of the gp120-CCR5Δ32 interaction, representing the minimal observed interaction in our reference set. It is important to emphasize that these thresholds serve specifically for comparative ranking within this screen; they indicate a candidate’s relative promise for further experimental validation, not its absolute biological significance.

Concurrently, all areas for HICGP interactions with background proteins underwent cluster analysis to identify patterns in HICGP involvement during viral entry. This analysis employed k-means clustering in IBM SPSS Statistics 27 software. Thus, the comparative analysis methodology involved data normalization to a unified scale relative to control interactions and statistical significance evaluation of identified contacts.

#### 4.2.4. Limitations of the Computational Approach

The methodology employed several intentional simplifications to enable a computationally tractable large-scale screening pipeline. The primary goal was the comparative ranking of a large set of candidate interactions, not the production of refined, atomic-resolution structural models. Consequently, to ensure feasibility and consistency across the screen, all AlphaFold models were used in their raw, unrefined output state. This means we deliberately abstained from:

- Post-processing refinement using tools like HADDOCK or RosettaDock, which would be computationally prohibitive at this scale but are essential for obtaining stable, energetically minimized complexes.
- Incorporation of key biophysical contexts, such as explicit lipid membranes, glycosylation of gp120, or physiological ionic conditions. Our models represent simplified binary interactions in vacuo.

These choices were necessary for feasibility but mean that the models should be viewed as rough scaffolds identifying potential interaction interfaces. The ranking provides a priority list for future, more focused experimental and computational validation.

## 5. Conclusions

The computational modeling and comparative ranking pipeline developed in this study enabled the systematic identification and prioritization of human proteins with the potential to physically interact with key components of the HIV attachment system. Our analysis highlights several promising candidates and mechanistic insights. Notably, the viral glycoprotein gp120 demonstrated a predicted capacity to interact with a broad spectrum of membrane receptors beyond the classical coreceptors, suggesting a more complex landscape of viral entry portals. It is important to note that the presented models, while useful for comparative ranking, represent simplifications that do not incorporate key biophysical contexts such as explicit lipid membranes, gp120 glycosylation, or physiological ionic conditions. Furthermore, we observed marked disparity in the binding potential of natural chemokine ligands, which may influence coreceptor selectivity and viral tropism. The identification of specific neuropeptides, such as PNOC and NPY, as potential interactors with the HIV entry machinery opens new avenues for investigating their role in infection and pathogenesis. Most importantly, the case of CCL27 emerged as a particularly compelling candidate, with a unique predicted capability for dual interaction with both the CCR5 coreceptor and viral gp120. Collectively, these findings provide a systematically ranked set of candidate targets and establish a robust computational framework for future studies of pathogen-host protein interactions, guiding subsequent in-depth bioinformatic analyses and prioritizing targets for experimental validation.

## Figures and Tables

**Table 1:** Model data for HCBGPs and CCR5-Δ32.

Protein	pTM	RS
CCR5	0.81	0.9
CCR5-del32	0.68	0.83
CXCR4	0.79	0.87
CCR2	0.75	0.87
CD4	0.69	0.79
gp120 HIV	0.78	0.81

**Table 2:** Parameters of HCBGP-HRP interaction models.

Protein	ipTM	pTM	RS
CCR5+gp120	0.34	**0.62**	0.45
CCR5-del32+gp120	0.17	0.46	0.31
CXCR4+gp120	0.49	**0.61**	**0.59**
CCR2+gp120	0.45	**0.6**	**0.57**
CD4+gp120	0.25	**0.54**	0.39

Note: Parameter values exceeding the threshold are indicated in bold.

**Table 3:** Contact quantification between background proteins and viral gp120 protein.

Protein	Contacts (Including Clashes)	Clashes	Hydrogen Bonds	Total
CCR5	210	12	26	224
CXCR4	364	22	25	367
CCR2	196	11	20	205
CD4	43	1	3	45
CCR5del32 *	129 *	12 *	9 *	126 *

* CCR5del32—model unreliable.

**Table 4:** Top-ranked HICGPs based on comparative Area analysis (values exceeding operational thresholds for prioritization are indicated in bold). For gp120 area, values exceeding the CCR5Δ32-based threshold are indicated in bold; values exceeding the CCR5+gp120 area threshold are indicated in italics.

HICGPs	Area				
	CCR5	CXCR4	CCR2	CD4	gp120
ACKR3	0.05125	0.038719	0.308	**1.832444**	***0.82976***
ADRA2C	0.03683	0.203651	0.143415	**1.024667**	***0.88063***
CCL2	**1.007589**	0.392861	**0.658537**	**0.526222**	0.079365
CCL25	**0.571339**	0.082888	**0.851902**	**0.416**	0.088889
CCL27	**0.468214**	0.184196	0.266341	**0.616222**	**0.678889**
CCL8	**1.2675**	0.135749	**0.924**	0.284444	0.115556
CCR7	0.09875	0.032044	**0.609415**	**0.91**	**0.744762**
CXCL12	**0.873393**	**0.591962**	0.468878	**0.439556**	0.094286
CXCL13	**0.652054**	0.308992	**0.715756**	**0.462222**	0.202381
CXCL2	**0.646875**	0.220272	**0.611122**	0.216	0.17127
CXCL3	**0.476741**	0.191771	**0.638927**	**0.654222**	0.25381
CXCR3	0.132054	0.140599	0.287171	**1.057778**	***1.05333***
CXCR6	0.181071	0.038256	0.139024	**0.756444**	***1.12***
GPR18	0.083571	0.146567	0.131707	0.296222	***0.9219***
HTR5A	0.083973	0.134877	0.026537	**0.662667**	***0.9673***
NPY1R	0.058125	0.056649	**0.729073**	**0.945778**	***1.25222***
NPY5R	0.078571	0.164905	**0.595463**	**0.412444**	***0.87659***
OPRK1	0.235804	0.107902	**0.637317**	**0.454667**	**0.761429**
PENK	0.252589	0.235177	**0.77522**	**0.746667**	0
PNOC	**0.498214**	**0.653297**	0.186439	**0.635556**	0.068571
PTGDR2	0.111339	0.078474	0.154537	**0.632**	***0.89532***
SSTR3	0.175	0.148583	0.19122	**0.489778**	***0.85317***

**Table 5:** Clustering results of HICGPs based on area parameter for their interaction models with HCBGPs and HRP. The clear separation into receptor and ligand clusters provides internal validation of our prioritization approach, demonstrating its ability to recapitulate fundamental biological categories.

Protein	Cluster	Distance (Arb. Units)
ACKR3	2	0.302
ADRA2A	2	0.341
ADRA2C	2	0.357
ANXA1	2	0.236
CCL19	2	0.323
CCL2	1	0.412
CCL20	1	0.375
CCL25	1	0.347
CCL27	2	0.349
CCL8	1	0.754
CCR10	2	0.220
CCR7	2	0.427
CCR9	2	0.247
CHRM2	2	0.322
CXCL12	1	0.408
CXCL13	1	0.148
CXCL2	1	0.107
CXCL3	1	0.224
CXCR3	2	0.493
CXCR5	2	0.138
CXCR6	2	0.571
FPR3	2	0.264
GALR2	2	0.273
GALR3	2	0.332
GNA13	2	0.417
GNAI1	2	0.450
GNAI2	1	0.469
gp120	1	0.348
GPER1	2	0.203
GPR18	2	0.379
HCAR3	2	0.429
HEBP1	2	0.172
HRH4	2	0.208
HTR1D	2	0.178
HTR1E	2	0.175
HTR1F	2	0.171
HTR5A	2	0.458
NPY	2	0.406
NPY1R	2	0.854
NPY5R	2	0.484
OPRK1	2	0.457
OXER1	2	0.263
OXGR1	2	0.507
PDYN	2	0.394
PENK	1	0.428
PNOC	1	0.549
PTGDR2	2	0.345
S1PR2	2	0.113
S1PR3	2	0.164
SST	2	0.270
SSTR1	2	0.292
SSTR3	2	0.295
SUCNR1	2	0.266
TAS2R14	2	0.089
TAS2R20	2	0.338
TAS2R5	2	0.203

Note: The ‘Distance’ value for each protein represents the Euclidean distance to the centroid of its assigned cluster. It is a measure of how well the object fits its cluster and should not be used to compare cohesion between different clusters, as clusters can vary significantly in their inherent size and density within the multidimensional feature space.

## Data Availability

The original contributions presented in this study are included in the article/Supplementary Materials. Further inquiries can be directed to the corresponding author.

## Supplementary Materials

The following supporting information can be downloaded at https://www.mdpi.com/article/10.3390/ijms262211209/s1.

## Abbreviations

The following abbreviations are used in this manuscript:

HICGPs	HIV-interacting candidate gene proteins
HCBGPs	HIV coreceptor background gene proteins
HRP	HIV reference protein
HIV	human immunodeficiency virus
gp120	glycoprotein 120
CCR5	C-C chemokine receptor type 5
CXCR4	C-X-C chemokine receptor type 4
CD4	cluster of differentiation 4
NCBI	National Center for Biotechnology Information
PPI	protein–protein interaction
pTM	predicted template modeling
ipTM	interface predicted template modeling
RS	ranking score

## Appendix A

**Table A1:** Model confidence metrics according to AlphaFold. Values exceeding the reliability threshold are indicated in bold.

HICGP	HCBGPs/HRP														
	CCR5			CXCR4			CCR2			CD4			gp120		
	ipTM	pTM	Score	ipTM	pTM	Score	ipTM	pTM	Score	ipTM	pTM	Score	ipTM	pTM	Score
ACKR3	0.15	0.46	0.28	0.16	0.46	0.29	0.26	0.5	0.41	0.24	0.47	0.38	0.3	**0.63**	0.41
ADRA2A	0.18	0.41	0.38	0.16	0.4	0.36	0.14	0.39	0.37	0.19	0.39	0.38	0.15	0.48	0.37
ADRA2C	0.14	0.41	0.33	0.17	0.42	0.37	0.14	0.4	0.35	0.37	0.42	**0.53**	0.17	**0.51**	0.38
ANXA1	0.13	0.48	0.25	0.25	**0.54**	0.37	0.18	0.49	0.31	0.12	0.45	0.25	0.54	**0.65**	**0.6**
CCL19	0.53	**0.7**	**0.68**	0.18	**0.62**	0.37	0.51	**0.65**	**0.67**	0.34	**0.57**	**0.5**	0.23	0.46	0.36
CCL2	**0.62**	**0.75**	**0.74**	0.37	**0.66**	**0.54**	**0.61**	**0.7**	**0.75**	0.13	**0.54**	0.32	0.11	0.43	0.25
CCL20	0.57	**0.74**	**0.71**	0.27	**0.65**	0.45	0.6	**0.69**	**0.76**	0.24	**0.56**	0.42	0.15	0.47	0.29
CCL25	**0.63**	**0.72**	**0.79**	0.17	**0.58**	0.39	0.55	**0.67**	**0.74**	0.14	**0.51**	0.36	0.14	0.41	0.32
CCL27	0.54	**0.73**	**0.69**	0.19	**0.61**	0.4	0.46	**0.65**	**0.65**	0.3	**0.56**	0.47	0.35	0.45	0.47
CCL8	**0.67**	**0.77**	**0.78**	0.29	**0.64**	0.47	**0.63**	**0.71**	**0.77**	0.12	**0.52**	0.32	0.11	0.43	0.26
CCR10	0.2	0.48	0.35	0.21	0.43	0.35	0.13	0.42	0.3	0.19	0.43	0.33	0.34	**0.61**	0.46
CCR7	0.13	0.43	0.28	0.13	0.42	0.28	0.15	0.42	0.31	0.22	0.44	0.35	0.35	**0.63**	0.46
CCR9	0.16	0.46	0.3	0.14	0.44	0.28	0.12	0.42	0.28	0.17	0.41	0.3	0.34	**0.61**	0.43
CHRM2	0.15	0.41	0.35	0.13	0.39	0.33	0.14	0.39	0.35	0.17	0.38	0.36	0.15	0.47	0.36
CXCL12	0.52	**0.73**	**0.67**	**0.66**	**0.76**	**0.79**	0.36	**0.66**	**0.54**	0.26	0.51	0.43	0.12	0.44	0.27
CXCL13	0.51	**0.72**	**0.67**	0.35	**0.65**	**0.54**	0.5	**0.68**	**0.67**	0.11	0.55	0.32	0.15	0.45	0.3
CXCL2	0.54	**0.73**	**0.69**	0.22	**0.63**	0.43	0.55	**0.69**	**0.72**	0.16	0.54	0.36	0.1	0.44	0.26
CXCL3	0.42	**0.68**	**0.59**	0.32	**0.65**	**0.51**	0.57	**0.7**	**0.74**	0.11	0.54	0.32	0.1	0.42	0.26
CXCR3	0.13	0.43	0.29	0.14	0.43	0.3	0.13	0.41	0.29	0.21	0.45	0.35	0.28	**0.6**	0.42
CXCR5	0.17	0.46	0.3	0.12	0.43	0.28	0.14	0.43	0.3	0.22	0.44	0.36	0.4	**0.6**	**0.51**
CXCR6	0.11	0.44	0.26	0.12	0.43	0.27	0.14	0.44	0.3	0.24	0.47	0.37	0.47	**0.64**	**0.56**
FPR3	0.24	**0.5**	0.37	0.21	0.45	0.33	0.34	0.46	0.45	0.48	0.49	**0.57**	0.49	**0.63**	**0.56**
GALR2	0.12	0.46	0.3	0.2	0.46	0.36	0.12	0.44	0.32	0.19	0.45	0.36	0.19	0.46	0.36
GALR3	0.15	0.46	0.3	0.13	0.44	0.29	0.15	0.44	0.32	0.21	0.44	0.36	0.13	0.44	0.29
GNA13	**0.61**	**0.65**	**0.68**	0.58	**0.63**	**0.65**	0.59	**0.63**	**0.68**	0.14	0.39	0.27	0.16	0.47	0.26
GNAI1	**0.74**	**0.69**	**0.79**	**0.71**	**0.67**	**0.76**	**0.67**	**0.67**	**0.74**	0.55	0.4	0.59	0.15	0.42	0.23
GNAI2	**0.75**	**0.7**	**0.79**	**0.72**	**0.68**	**0.77**	**0.68**	**0.67**	**0.75**	0.34	**0.5**	0.45	0.13	0.43	0.21
GPER1	0.28	0.45	0.41	0.14	0.42	0.29	0.5	**0.6**	**0.63**	0.19	0.44	0.34	0.21	**0.54**	0.34
GPR18	0.14	0.48	0.26	0.2	0.46	0.33	0.14	0.44	0.3	0.18	0.44	0.31	0.35	**0.58**	0.44
HCAR3	0.16	0.44	0.31	0.12	0.43	0.28	0.18	0.44	0.35	0.18	0.43	0.33	0.18	**0.56**	0.33
HEBP1	0.29	**0.61**	0.42	0.25	**0.57**	0.37	0.16	**0.53**	0.32	0.12	0.48	0.28	0.26	**0.54**	0.33
HRH4	0.13	0.44	0.29	0.15	0.44	0.32	0.12	0.41	0.3	0.17	0.4	0.32	0.13	**0.51**	0.31
HTR1D	0.17	0.47	0.33	0.18	0.44	0.34	0.14	0.44	0.33	0.18	0.41	0.34	0.27	**0.54**	0.42
HTR1E	0.17	0.46	0.32	0.14	0.44	0.3	0.18	0.46	0.35	0.37	0.53	0.51	0.21	**0.56**	0.36
HTR1F	0.13	0.44	0.28	0.18	0.46	0.32	0.14	0.44	0.3	0.25	0.42	0.38	0.22	**0.56**	0.35
HTR5A	0.17	0.47	0.33	0.13	0.42	0.3	0.16	0.43	0.34	0.25	0.45	0.42	0.31	**0.58**	0.44
NPY	0.2	**0.65**	0.43	0.37	**0.67**	**0.51**	0.2	**0.6**	0.45	0.16	**0.56**	0.39	0.11	0.48	0.34
NPY1R	0.16	0.45	0.31	0.18	0.46	0.33	0.34	0.46	0.47	0.23	0.43	0.38	0.33	**0.58**	0.46
NPY5R	0.15	0.42	0.32	0.17	0.42	0.34	0.22	0.42	0.39	0.16	0.38	0.32	0.31	**0.56**	0.47
OPRK1	0.23	0.49	0.38	0.15	0.43	0.3	0.23	0.44	0.39	0.16	0.4	0.31	0.27	**0.59**	0.41
OXER1	0.15	0.45	0.32	0.21	0.44	0.36	0.13	0.42	0.3	0.22	0.44	0.38	0.29	**0.58**	0.42
OXGR1	0.2	**0.5**	0.33	0.13	0.45	0.26	0.22	0.48	0.36	0.17	0.42	0.3	0.11	**0.59**	0.24
PDYN	0.21	**0.51**	0.47	0.23	**0.51**	**0.51**	0.15	0.48	0.45	0.12	0.47	0.4	0.17	0.36	0.45
PENK	0.18	**0.51**	0.46	0.37	**0.53**	**0.63**	0.3	**0.5**	**0.58**	0.49	**0.52**	0.7	0.08	0.36	0.39
PNOC	0.15	**0.57**	0.4	0.55	**0.63**	**0.72**	0.15	**0.53**	0.42	0.31	**0.53**	**0.52**	0.12	0.41	0.36
PTGDR2	0.14	0.45	0.29	0.17	0.47	0.32	0.16	0.45	0.33	0.21	0.46	0.36	0.14	**0.57**	0.29
S1PR2	0.24	0.49	0.37	0.18	0.47	0.32	0.22	0.48	0.36	0.2	0.44	0.34	0.2	**0.58**	0.33
S1PR3	0.19	0.47	0.34	0.17	0.43	0.32	0.29	**0.5**	0.44	0.18	0.43	0.33	0.24	**0.58**	0.38
SST	0.25	**0.66**	**0.52**	0.21	**0.62**	0.48	0.22	**0.61**	0.49	0.14	**0.55**	0.4	0.31	**0.53**	**0.56**
SSTR1	0.19	0.44	0.35	0.16	0.42	0.32	0.19	0.41	0.36	0.17	0.41	0.33	0.24	**0.56**	0.39
SSTR3	0.18	0.44	0.35	0.25	0.43	0.41	0.16	0.4	0.35	0.2	0.41	0.38	0.26	**0.55**	0.43
SUCNR1	0.17	0.49	0.29	0.18	0.49	0.31	0.25	**0.51**	0.38	0.2	0.47	0.33	0.29	**0.66**	0.39
TAS2R14	0.27	**0.53**	0.38	0.12	0.45	0.26	0.22	0.45	0.35	0.21	0.44	0.33	0.33	**0.62**	0.41
TAS2R20	0.12	0.46	0.24	0.2	0.45	0.31	0.14	0.46	0.27	0.28	**0.5**	0.38	0.27	**0.62**	0.35
TAS2R5	0.15	0.48	0.27	0.13	0.46	0.27	0.12	0.45	0.26	0.22	0.44	0.33	0.15	**0.52**	0.25

## Appendix B

**Table A2:** Manual structural classification of predicted protein complexes. Structural classification symbols: c—contact; ?—ambiguous; *—membrane penetration; **—side-by-side receptors; ***—vertical stacking.

Proteins	Visual Assessment				
	CCR5	CXCR4	CCR2	CD4	gp120
ACKR3	**	**	***	c	c
ADRA2A	**	**	**	?	c
ADRA2C	**	**	**	*	c
ANXA1	c	c	c	c	c
CCL19	c	c	c	c	c
CCL2	c	c	c	c	c
CCL20	c	c	c	c	c
CCL25	c	c	c	c	c
CCL27	c	c	c	c	c
CCL8	c	c	c	c	c
CCR10	**	***	**	*	c
CCR7	**	**	**	?	c
CCR9	**	**	**	*	c
CHRM2	**	**	**	***	c
CXCL12	c	c	c	?	c
CXCL13	c	c	c	c	c
CXCL2	c	c	c	c	c
CXCL3	c	c	c	c	c
CXCR3	**	**	**	c	c
CXCR5	**	**	**	*	c
CXCR6	**	**	**	*	c
FPR3	**	**	**	?	c
GALR2	**	**	**	*	c
GALR3	**	**	**	c	c
GNA13	c	c	c	c	c
GNAI1	c	c	c	*	c
GNAI2	c	c	c	c	c
GPER1	**	**	**	*	c
GPR18	**	**	**	*	c
HCAR3	**	**	**	*	c
HEBP1	c	c	c	c	c
HRH4	**	**	**	*	c
HTR1D	**	***	**	*	c
HTR1E	**	**	**	*	c
HTR1F	**	**	**	*	c
HTR5A	**	**	**	c	c
NPY	c	c	c	c	c
NPY1R	**	**	**	c	c
NPY5R	**	**	**	*	c
OPRK1	***	**	**	*	c
OXER1	**	**	**	c	c
OXGR1	**	**	**	*	c
PDYN	c	c	?	?	c
PENK	c	c	*	c	?
PNOC	*	*	c	c	c
PTGDR2	**	**	**	*	c
S1PR2	**	**	**	*	c
S1PR3	**	**	**	*	c
SST	*	c	c	*	c
SSTR1	**	**	**	*	c
SSTR3	**	**	**	c	c
SUCNR1	**	**	**	*	c
TAS2R14	**	**	**	*	c
TAS2R20	**	**	**	?	c
TAS2R5	**	**	**	*	c

## Appendix C

**Table A3:** Contact, clash, and hydrogen bond metrics for HICGP interactions with HCBGPs/HRP, normalized against background interactions.

Proteins	Contacts					Clashes					Hydrogen Bonds					Total				
	CCR5	CXCR4	CCR2	CD4	gp120	CCR5	CXCR4	CCR2	CD4	gp120	CCR5	CXCR4	CCR2	CD4	gp120	CCR5	CXCR4	CCR2	CD4	gp120
ACKR3	45	55	154	236	265	4	6	5	32	26	0	0	5	13	16	41	49	154	217	255
ADRA2A	167	160	76	73	99	17	20	12	4	9	8	7	0	3	2	158	147	64	72	92
ADRA2C	25	250	99	102	337	1	52	15	18	57	1	4	0	3	12	25	202	84	87	292
ANXA1	113	122	53	94	83	11	13	4	9	6	3	10	3	4	5	105	119	52	89	82
CCL19	101	129	119	30	115	7	16	6	0	4	8	2	12	3	10	102	115	125	33	121
CCL2	302	277	185	79	40	23	25	12	7	2	26	15	7	2	2	305	267	180	74	40
CCL20	123	112	99	68	126	6	3	4	8	10	11	7	9	4	9	128	116	104	64	125
CCL25	154	80	236	67	35	6	7	15	16	0	14	5	15	1	0	162	78	236	52	35
CCL27	141	171	78	60	189	5	12	2	2	20	16	10	8	1	13	152	169	84	59	182
CCL8	373	106	261	38	53	33	5	26	3	3	24	5	11	5	6	364	106	246	40	56
CCR10	50	223	40	97	161	5	16	3	8	14	0	13	0	7	19	45	220	37	96	166
CCR7	78	45	485	136	206	3	5	98	21	9	4	2	16	2	7	79	42	403	117	204
CCR9	127	115	105	82	227	18	11	11	4	11	1	5	1	3	14	110	109	95	81	230
CHRM2	62	89	31	22	114	6	17	6	2	9	4	0	0	1	6	60	72	25	21	111
CXCL12	304	255	197	47	45	30	7	22	2	5	18	27	3	1	4	292	275	178	46	44
CXCL13	219	214	214	68	91	12	19	10	8	10	11	15	15	5	4	218	210	219	65	85
CXCL2	211	192	172	25	91	8	11	9	1	10	7	7	11	3	2	210	188	174	27	83
CXCL3	180	137	166	112	142	8	7	5	21	21	9	8	16	1	2	181	138	177	92	123
CXCR3	114	212	229	137	318	13	41	34	3	28	1	1	8	2	26	102	172	203	136	316
CXCR5	66	390	135	163	154	7	116	8	11	12	2	4	1	6	13	61	278	128	158	155
CXCR6	190	57	99	92	256	39	5	7	8	17	5	0	3	8	13	156	52	95	92	252
FPR3	65	151	217	220	159	3	13	13	18	11	1	7	6	6	8	63	145	210	208	156
GALR2	188	38	65	88	126	19	2	5	4	6	6	1	2	2	7	175	37	62	86	127
GALR3	89	72	58	112	122	9	7	2	13	7	1	3	1	5	5	81	68	57	104	120
GNA13	59	61	87	31	70	2	2	5	2	2	5	6	5	1	6	62	65	87	30	74
GNAI1	54	71	75	47	69	0	0	1	1	5	5	9	13	6	6	59	80	87	52	70
GNAI2	61	69	87	39	41	0	0	1	0	0	7	12	10	4	1	68	81	96	43	42
GPER1	138	54	64	120	282	10	6	1	12	26	9	1	3	6	9	137	49	66	114	265
GPR18	79	178	95	45	295	7	20	7	4	43	0	5	2	2	12	72	163	90	43	264
HCAR3	165	70	109	50	66	16	1	14	3	8	9	3	2	2	0	158	72	97	49	58
HEBP1	86	96	103	158	152	9	9	5	33	4	3	3	2	4	10	80	90	100	129	158
HRH4	43	120	90	90	192	3	6	9	6	27	1	5	4	3	4	41	119	85	87	169
HTR1D	73	284	125	78	201	7	33	14	9	11	6	14	1	2	11	72	265	112	71	201
HTR1E	93	131	125	118	146	7	8	15	5	11	1	3	3	9	4	87	126	113	122	139
HTR1F	30	169	83	65	238	3	52	5	7	25	3	0	4	2	12	30	117	82	60	225
HTR5A	60	209	16	65	270	7	47	0	2	12	4	3	0	8	19	57	165	16	71	277
NPY	138	175	108	66	68	11	10	2	18	1	6	11	6	2	5	133	176	112	50	72
NPY1R	41	67	364	109	358	0	6	58	7	30	1	2	12	10	15	42	63	318	112	343
NPY5R	58	205	330	59	246	4	29	32	4	24	1	2	15	3	13	55	178	313	58	235
OPRK1	143	139	392	66	232	10	10	67	1	10	6	3	10	1	12	139	132	335	66	234
OXER1	53	286	129	135	246	4	44	18	16	26	0	10	3	6	11	49	252	114	125	231
OXGR1	69	60	77	70	41	4	2	5	4	2	1	0	0	2	2	66	58	72	68	41
PDYN	65	172	122	39	57	2	16	6	1	7	4	12	9	2	3	67	168	125	40	53
PENK	113	138	313	41	0	2	11	45	1	0	12	10	6	8	0	123	137	274	48	0
PNOC	298	326	90	51	23	34	20	4	0	0	15	27	5	4	1	279	333	91	55	24
PTGDR2	99	95	101	84	491	13	8	8	9	107	0	3	3	4	5	86	90	96	79	389
S1PR2	32	199	114	80	246	2	28	5	5	26	2	2	2	2	10	32	173	111	77	230
S1PR3	55	107	135	97	134	4	9	17	9	8	3	4	10	0	14	54	102	128	88	140
SST	186	125	112	30	129	20	13	8	5	8	11	8	8	1	12	177	120	112	26	133
SSTR1	119	98	230	72	110	7	8	25	11	11	3	6	5	1	1	115	96	210	62	100
SSTR3	116	139	122	53	272	10	15	11	0	29	6	9	1	5	7	112	133	112	58	250
SUCNR1	78	80	98	68	101	6	10	6	5	3	0	0	0	3	5	72	70	92	66	103
TAS2R14	52	171	192	88	176	1	30	19	5	13	1	6	2	2	5	52	147	175	85	168
TAS2R20	126	162	115	173	84	10	11	10	5	4	2	3	5	5	6	118	154	110	173	86
TAS2R5	174	70	165	71	207	21	9	21	11	16	1	0	1	5	4	154	61	145	65	195

**Table A4:** Interaction values for HICGPs with HCBGPs/gp120 relative to background interactions.

Proteins	Normalized Interaction Values				
	CCR5	CXCR4	CCR2	CD4	gp120
ACKR3	0.18304	0.13351	0.75122	4.82222	2.02381
ADRA2A	0.70536	0.40054	0.31220	1.60000	0.73016
ADRA2C	0.11161	0.55041	0.40976	1.93333	2.31746
ANXA1	0.46875	0.32425	0.25366	1.97778	0.65079
CCL19	0.45536	0.31335	0.60976	0.73333	0.96032
CCL2	1.36161	0.72752	0.87805	1.64444	0.31746
CCL20	0.57143	0.31608	0.50732	1.42222	0.99206
CCL25	0.72321	0.21253	1.15122	1.15556	0.27778
CCL27	0.67857	0.46049	0.40976	1.31111	1.44444
CCL8	1.62500	0.28883	1.20000	0.88889	0.44444
CCR10	0.20089	0.59946	0.18049	2.13333	1.31746
CCR7	0.35268	0.11444	1.96585	2.60000	1.61905
CCR9	0.49107	0.29700	0.46341	1.80000	1.82540
CHRM2	0.26786	0.19619	0.12195	0.46667	0.88095
CXCL12	1.30357	0.74932	0.86829	1.02222	0.34921
CXCL13	0.97321	0.57221	1.06829	1.44444	0.67460
CXCL2	0.93750	0.51226	0.84878	0.60000	0.65873
CXCL3	0.80804	0.37602	0.86341	2.04444	0.97619
CXCR3	0.45536	0.46866	0.99024	3.02222	2.50794
CXCR5	0.27232	0.75749	0.62439	3.51111	1.23016
CXCR6	0.69643	0.14169	0.46341	2.04444	2.00000
FPR3	0.28125	0.39510	1.02439	4.62222	1.23810
GALR2	0.78125	0.10082	0.30244	1.91111	1.00794
GALR3	0.36161	0.18529	0.27805	2.31111	0.95238
GNA13	0.27679	0.17711	0.42439	0.66667	0.58730
GNAI1	0.26339	0.21798	0.42439	1.15556	0.55556
GNAI2	0.30357	0.22071	0.46829	0.95556	0.33333
GPER1	0.61161	0.13351	0.32195	2.53333	2.10317
GPR18	0.32143	0.44414	0.43902	0.95556	2.09524
HCAR3	0.70536	0.19619	0.47317	1.08889	0.46032
HEBP1	0.35714	0.24523	0.48780	2.86667	1.25397
HRH4	0.18304	0.32425	0.41463	1.93333	1.34127
HTR1D	0.32143	0.72207	0.54634	1.57778	1.59524
HTR1E	0.38839	0.34332	0.55122	2.71111	1.10317
HTR1F	0.13393	0.31880	0.40000	1.33333	1.78571
HTR5A	0.25446	0.44959	0.07805	1.57778	2.19841
NPY	0.59375	0.47956	0.54634	1.11111	0.57143
NPY1R	0.18750	0.17166	1.55122	2.48889	2.72222
NPY5R	0.24554	0.48501	1.52683	1.28889	1.86508
OPRK1	0.62054	0.35967	1.63415	1.46667	1.85714
OXER1	0.21875	0.68665	0.55610	2.77778	1.83333
OXGR1	0.29464	0.15804	0.35122	1.51111	0.32540
PDYN	0.29911	0.45777	0.60976	0.88889	0.42063
PENK	0.54911	0.37330	1.33659	1.06667	-
PNOC	1.24554	0.90736	0.44390	1.22222	0.19048
PTGDR2	0.38393	0.24523	0.46829	1.75556	3.08730
S1PR2	0.14286	0.47139	0.54146	1.71111	1.82540
S1PR3	0.24107	0.27793	0.62439	1.95556	1.11111
SST	0.79018	0.32698	0.54634	0.57778	1.05556
SSTR1	0.51339	0.26158	1.02439	1.37778	0.79365
SSTR3	0.50000	0.36240	0.54634	1.28889	1.98413
SUCNR1	0.32143	0.19074	0.44878	1.46667	0.81746
TAS2R14	0.23214	0.40054	0.85366	1.88889	1.33333
TAS2R20	0.52679	0.41962	0.53659	3.84444	0.68254
TAS2R5	0.68750	0.16621	0.70732	1.44444	1.54762

## Appendix D

**Table A5:** HCBGP area thresholds.

HCBGP	Area	Threshold (95% Area)
CCR5	0.45	0.4275
CXCR4	0.59	0.5605
CCR2	0.57	0.5415
CD4	0.39	0.3705
CCR5del32	0.31	

## Appendix E

**Table A6:** Area values for HICGPs. For AlphaFold Score, values exceeding thresholds are indicated in bold. For HCBG Area, values exceeding Threshold are indicated in bold. For gp120 area, values exceeding the CCR5Δ32-based threshold are indicated in bold; values exceeding the CCR5+gp120 area threshold are indicated in italics.

HICGPs	Alphafold Score					Contacts Normal					Area				
	CCR5	CXCR4	CCR2	CD4	gp120	CCR5	CXCR4	CCR2	CD4	gp120	CCR5	CXCR4	CCR2	CD4	gp120
ACKR3	0.28	0.29	0.41	0.38	0.41	0.18304	0.13351	0.75122	4.82222	2.02381	0.05125	0.03872	0.30800	**1.83244**	***0.82976***
ADRA2A	0.38	0.36	0.37	0.38	0.37	0.70536	0.40054	0.31220	1.60000	0.73016	0.26804	0.14420	0.11551	**0.60800**	0.27016
ADRA2C	0.33	0.37	0.35	**0.53**	0.38	0.11161	0.55041	0.40976	1.93333	2.31746	0.03683	0.20365	0.14341	**1.02467**	***0.88063***
ANXA1	0.25	0.37	0.31	0.25	**0.6**	0.46875	0.32425	0.25366	1.97778	0.65079	0.11719	0.11997	0.07863	**0.49444**	**0.39048**
CCL19	**0.68**	0.37	**0.67**	**0.5**	0.36	0.45536	0.31335	0.60976	0.73333	0.96032	0.30964	0.11594	0.40854	0.36667	**0.34571**
CCL2	**0.74**	**0.54**	**0.75**	0.32	0.25	1.36161	0.72752	0.87805	1.64444	0.31746	**1.00759**	0.39286	**0.65854**	**0.52622**	0.07937
CCL20	**0.71**	0.45	**0.76**	0.42	0.29	0.57143	0.31608	0.50732	1.42222	0.99206	0.40571	0.14223	0.38556	**0.59733**	0.28770
CCL25	**0.79**	0.39	**0.74**	0.36	0.32	0.72321	0.21253	1.15122	1.15556	0.27778	**0.57134**	0.08289	**0.85190**	**0.41600**	0.08889
CCL27	**0.69**	0.4	**0.65**	0.47	0.47	0.67857	0.46049	0.40976	1.31111	1.44444	**0.46821**	0.18420	0.26634	**0.61622**	0.67889
CCL8	**0.78**	0.47	**0.77**	0.32	0.26	1.62500	0.28883	1.20000	0.88889	0.44444	**1.26750**	0.13575	**0.92400**	0.28444	0.11556
CCR10	0.35	0.35	0.3	0.33	0.46	0.20089	0.59946	0.18049	2.13333	1.31746	0.07031	0.20981	0.05415	**0.70400**	0.60603
CCR7	0.28	0.28	0.31	0.35	0.46	0.35268	0.11444	1.96585	2.60000	1.61905	0.09875	0.03204	**0.60941**	**0.91000**	0.74476
CCR9	0.3	0.28	0.28	0.3	0.43	0.49107	0.29700	0.46341	1.80000	1.82540	0.14732	0.08316	0.12976	**0.54000**	0.78492
CHRM2	0.35	0.33	0.35	0.36	0.36	0.26786	0.19619	0.12195	0.46667	0.88095	0.09375	0.06474	0.04268	0.16800	0.31714
CXCL12	**0.67**	**0.79**	**0.54**	0.43	0.27	1.30357	0.74932	0.86829	1.02222	0.34921	**0.87339**	**0.59196**	0.46888	**0.43956**	0.09429
CXCL13	**0.67**	**0.54**	**0.67**	0.32	0.3	0.97321	0.57221	1.06829	1.44444	0.67460	**0.65205**	0.30899	**0.71576**	**0.46222**	0.20238
CXCL2	**0.69**	**0.43**	**0.72**	0.36	0.26	0.93750	0.51226	0.84878	0.60000	0.65873	**0.64688**	0.22027	**0.61112**	0.21600	0.17127
CXCL3	**0.59**	**0.51**	**0.74**	0.32	0.26	0.80804	0.37602	0.86341	2.04444	0.97619	**0.47674**	0.19177	**0.63893**	**0.65422**	0.25381
CXCR3	0.29	0.3	0.29	0.35	0.42	0.45536	0.46866	0.99024	3.02222	2.50794	0.13205	0.14060	0.28717	**1.05778**	*1.05333*
CXCR5	0.3	0.28	0.3	0.36	**0.51**	0.27232	0.75749	0.62439	3.51111	1.23016	0.08170	0.21210	0.18732	**1.26400**	0.62738
CXCR6	0.26	0.27	0.3	0.37	**0.56**	0.69643	0.14169	0.46341	2.04444	2.00000	0.18107	0.03826	0.13902	**0.75644**	*1.12000*
FPR3	0.37	0.33	0.45	**0.57**	**0.56**	0.28125	0.39510	1.02439	4.62222	1.23810	0.10406	0.13038	0.46098	**2.63467**	0.69333
GALR2	0.3	0.36	0.32	0.36	0.36	0.78125	0.10082	0.30244	1.91111	1.00794	0.23438	0.03629	0.09678	**0.68800**	**0.36286**
GALR3	0.3	0.29	0.32	0.36	0.29	0.36161	0.18529	0.27805	2.31111	0.95238	0.10848	0.05373	0.08898	**0.83200**	0.27619
GNA13	**0.68**	**0.65**	**0.68**	0.27	0.26	0.27679	0.17711	0.42439	0.66667	0.58730	0.18821	0.11512	0.28859	0.18000	0.15270
GNAI1	**0.79**	**0.76**	**0.74**	**0.59**	0.23	0.26339	0.21798	0.42439	1.15556	0.55556	0.20808	0.16567	0.31405	**0.68178**	0.12778
GNAI2	**0.79**	**0.77**	**0.75**	0.45	0.21	0.30357	0.22071	0.46829	0.95556	0.33333	0.23982	0.16995	0.35122	**0.43000**	0.07000
GPER1	0.41	0.29	**0.63**	0.34	0.34	0.61161	0.13351	0.32195	2.53333	2.10317	0.25076	0.03872	0.20283	**0.86133**	**0.71508**
GPR18	0.26	0.33	0.3	0.31	0.44	0.32143	0.44414	0.43902	0.95556	2.09524	0.08357	0.14657	0.13171	0.29622	***0.92190***
HCAR3	0.31	0.28	0.35	0.33	0.33	0.70536	0.19619	0.47317	1.08889	0.46032	0.21866	0.05493	0.16561	0.35933	0.15190
HEBP1	0.42	0.37	0.32	0.28	0.33	0.35714	0.24523	0.48780	2.86667	1.25397	0.15000	0.09074	0.15610	**0.80267**	**0.41381**
HRH4	0.29	0.32	0.3	0.32	0.31	0.18304	0.32425	0.41463	1.93333	1.34127	0.05308	0.10376	0.12439	**0.61867**	**0.41579**
HTR1D	0.33	0.34	0.33	0.34	0.42	0.32143	0.72207	0.54634	1.57778	1.59524	0.10607	0.24550	0.18029	**0.53644**	**0.67000**
HTR1E	0.32	0.3	0.35	**0.51**	0.36	0.38839	0.34332	0.55122	2.71111	1.10317	0.12429	0.10300	0.19293	**1.38267**	**0.39714**
HTR1F	0.28	0.32	0.3	0.38	0.35	0.13393	0.31880	0.40000	1.33333	1.78571	0.03750	0.10202	0.12000	**0.50667**	**0.62500**
HTR5A	0.33	0.3	0.34	0.42	0.44	0.25446	0.44959	0.07805	1.57778	2.19841	0.08397	0.13488	0.02654	**0.66267**	***0.96730***
NPY	0.43	**0.51**	0.45	0.39	0.34	0.59375	0.47956	0.54634	1.11111	0.57143	0.25531	0.24458	0.24585	**0.43333**	0.19429
NPY1R	0.31	0.33	0.47	0.38	0.46	0.18750	0.17166	1.55122	2.48889	2.72222	0.05813	0.05665	**0.72907**	**0.94578**	***1.25222***
NPY5R	0.32	0.34	0.39	0.32	0.47	0.24554	0.48501	1.52683	1.28889	1.86508	0.07857	0.16490	**0.59546**	**0.41244**	***0.87659***
OPRK1	0.38	0.3	0.39	0.31	0.41	0.62054	0.35967	1.63415	1.46667	1.85714	0.23580	0.10790	**0.63732**	**0.45467**	**0.76143**
OXER1	0.32	0.36	0.3	0.38	0.42	0.21875	0.68665	0.55610	2.77778	1.83333	0.07000	0.24719	0.16683	**1.05556**	**0.77000**
OXGR1	0.33	0.26	0.36	0.3	0.24	0.29464	0.15804	0.35122	1.51111	0.32540	0.09723	0.04109	0.12644	**0.45333**	0.07810
PDYN	0.47	**0.51**	0.45	0.4	0.45	0.29911	0.45777	0.60976	0.88889	0.42063	0.14058	0.23346	0.27439	0.35556	0.18929
PENK	0.46	**0.63**	**0.58**	**0.7**	0.39	0.54911	0.37330	1.33659	1.06667	0.00000	0.25259	0.23518	**0.77522**	**0.74667**	0.00000
PNOC	0.4	**0.72**	0.42	**0.52**	0.36	1.24554	0.90736	0.44390	1.22222	0.19048	**0.49821**	**0.65330**	0.18644	**0.63556**	0.06857
PTGDR2	0.29	0.32	0.33	0.36	0.29	0.38393	0.24523	0.46829	1.75556	3.08730	0.11134	0.07847	0.15454	**0.63200**	***0.89532***
S1PR2	0.37	0.32	0.36	0.34	0.33	0.14286	0.47139	0.54146	1.71111	1.82540	0.05286	0.15084	0.19493	**0.58178**	**0.60238**
S1PR3	0.34	0.32	0.44	0.33	0.38	0.24107	0.27793	0.62439	1.95556	1.11111	0.08196	0.08894	0.27473	**0.64533**	**0.42222**
SST	**0.52**	0.48	0.49	0.4	**0.56**	0.79018	0.32698	0.54634	0.57778	1.05556	0.41089	0.15695	0.26771	0.23111	**0.59111**
SSTR1	0.35	0.32	0.36	0.33	0.39	0.51339	0.26158	1.02439	1.37778	0.79365	0.17969	0.08371	0.36878	**0.45467**	0.30952
SSTR3	0.35	0.41	0.35	0.38	0.43	0.50000	0.36240	0.54634	1.28889	1.98413	0.17500	0.14858	0.19122	**0.48978**	***0.85317***
SUCNR1	0.29	0.31	0.38	0.33	0.39	0.32143	0.19074	0.44878	1.46667	0.81746	0.09321	0.05913	0.17054	**0.48400**	**0.31881**
TAS2R14	0.38	0.26	0.35	0.33	0.41	0.23214	0.40054	0.85366	1.88889	1.33333	0.08821	0.10414	0.29878	**0.62333**	**0.54667**
TAS2R20	0.24	0.31	0.27	0.38	0.35	0.52679	0.41962	0.53659	3.84444	0.68254	0.12643	0.13008	0.14488	**1.46089**	0.23889
TAS2R5	0.27	0.27	0.26	0.33	0.25	0.68750	0.16621	0.70732	1.44444	1.54762	0.18563	0.04488	0.18390	**0.47667**	**0.38690**

## Appendix F

**Table A7:** Proteins nomenclature and GenBank numbers used in the study.

Proteins Abbreviation	GenBank Number	Proteins Name
ACKR3	XP_054199043.1	atypical chemokine receptor 3 isoform X1 [*Homo sapiens*]
ADRA2A	NP_000672.3	alpha-2A adrenergic receptor [*Homo sapiens*]
ADRA2C	ABY87522.1	adrenergic alpha-2C-receptor [*Homo sapiens*]
ANXA1	XP_054218828.1	annexin A1 isoform X1 [*Homo sapiens*]
CCL19	EAW58416.1	chemokine (C-C motif) ligand 19 [*Homo sapiens*]
CCL2	AAP35993.1	chemokine (C-C motif) ligand 2 [*Homo sapiens*]
CCL20	AAH20698.1	Chemokine (C-C motif) ligand 20 [*Homo sapiens*]
CCL25	AAI44464.1	CCL25 protein [*Homo sapiens*]
CCL27	EAW58421.1	chemokine (C-C motif) ligand 27 [*Homo sapiens*]
CCL8	AAI26243.1	Chemokine (C-C motif) ligand 8 [*Homo sapiens*]
CCR10	NP_057686.2	C-C chemokine receptor type 10 [*Homo sapiens*]
CCR7	EAW60669.1	chemokine (C-C motif) receptor 7 [*Homo sapiens*]
CCR9	NP_001373376.1	C-C chemokine receptor type 9 isoform A [*Homo sapiens*]
CHRM2	NP_001365901.1	muscarinic acetylcholine receptor M2 [*Homo sapiens*]
CXCL12	AAV49999.1	chemokine (C-X-C motif) ligand 12 (stromal cell-derived factor 1) [*Homo sapiens*]
CXCL13	AAH12589.1	Chemokine (C-X-C motif) ligand 13 [*Homo sapiens*]
CXCL2	CAG46968.1	CXCL2 [*Homo sapiens*]
CXCL3	AAH65743.1	Chemokine (C-X-C motif) ligand 3 [*Homo sapiens*]
CXCR3	NP_001495.1	C-X-C chemokine receptor type 3 isoform 1 [*Homo sapiens*]
CXCR5	XP_054225616.1	C-X-C chemokine receptor type 5 isoform X1 [*Homo sapiens*]
CXCR6	NP_001373364.1	C-X-C chemokine receptor type 6 [*Homo sapiens*]
FPR3	XP_054176392.1	N-formyl peptide receptor 3 isoform X1 [*Homo sapiens*]
GALR2	XP_054173638.1	galanin receptor type 2 isoform X1 [*Homo sapiens*]
GALR3	EAW60191.1	galanin receptor 3 [*Homo sapiens*]
GNA13	NP_006563.2	guanine nucleotide-binding protein subunit alpha-13 isoform 1 [*Homo sapiens*]
GNAI1	AAM12619.1	guanine nucleotide binding protein alpha i1 [*Homo sapiens*]
GNAI2	XP_054202176.1	guanine nucleotide-binding protein G(i) subunit alpha-2 isoform X1 [*Homo sapiens*]
GPER1	EAL23938.1	G protein-coupled receptor 30 [*Homo sapiens*]
GPR18	AFF59486.1	G protein-coupled receptor 18 [*Homo sapiens*]
HCAR3	APT70330.1	hydroxycarboxylic acid receptor 3 [*Homo sapiens*]
HEBP1	EAW96296.1	heme binding protein 1 isoform CRA_a [*Homo sapiens*]
HRH4	ACA05997.1	histamine H4 receptor [*Homo sapiens*]
HTR1D	EAW95037.1	5-hydroxytryptamine (serotonin) receptor 1D [*Homo sapiens*]
HTR1E	AAH69751.1	HTR1E protein [*Homo sapiens*]
HTR1F	NP_001309138.1	5-hydroxytryptamine receptor 1F [*Homo sapiens*]
HTR5A	EAX04526.1	5-hydroxytryptamine (serotonin) receptor 5A [*Homo sapiens*]
NPY	AAA59944.1	neuropeptide Y [*Homo sapiens*]
NPY1R	EAX04841.1	neuropeptide Y receptor Y1 [*Homo sapiens*]
NPY5R	XP_054206099.1	neuropeptide Y receptor type 5 isoform X1 [*Homo sapiens*]
OPRK1	EAW86723.1	opioid receptor kappa 1 [*Homo sapiens*]
OXER1	NP_683765.2	oxoeicosanoid receptor 1 [*Homo sapiens*]
OXGR1	NP_001333126.1	2-oxoglutarate receptor 1 [*Homo sapiens*]
PDYN	XP_054179481.1	proenkephalin-B isoform X1 [*Homo sapiens*]
PENK	CAG46607.1	PENK [*Homo sapiens*]
PNOC	AAV38141.1	prepronociceptin [*Homo sapiens*]
PTGDR2	NP_004769.2	prostaglandin D2 receptor 2 [*Homo sapiens*]
S1PR2	NP_004221.3	sphingosine 1-phosphate receptor 2 [*Homo sapiens*]
S1PR3	NP_001382777.1	sphingosine 1-phosphate receptor 3 [*Homo sapiens*]
SST	AAH32625.1	Somatostatin [*Homo sapiens*]
SSTR1	EAW65836.1	somatostatin receptor 1 [*Homo sapiens*]
SSTR3	EAW60148.1	somatostatin receptor 3 [*Homo sapiens*]
SUCNR1	ABY87909.1	succinate receptor 1 [*Homo sapiens*]
TAS2R14	EAW96222.1	taste receptor, type 2, member 14 [*Homo sapiens*]
TAS2R20	NP_795370.2	taste receptor type 2 member 20 [*Homo sapiens*]
TAS2R5	EAW83984.1	taste receptor type 2 member 5 [*Homo sapiens*]
CCR5	NP_001381712.1	C-C chemokine receptor type 5 [*Homo sapiens*]
CXCR4	EAX11616.1	chemokine (C-X-C motif) receptor 4 [*Homo sapiens*]
CD4	QDC22486.1	CD4 [*Homo sapiens*]
CCR2	AAI26453.1	Chemokine (C-C motif) receptor 2 [*Homo sapiens*]
gp120	AWU79409.1	envelope glycoprotein, partial [Human immunodeficiency virus 1]
